# Oncogenic HPV among HIV infected female population in West Bengal, India

**DOI:** 10.1186/1471-2334-11-72

**Published:** 2011-03-22

**Authors:** Kamalesh Sarkar, Reshmi Pal, Baishali Bal, Bibhuti Saha, Subhasish Bhattacharya, Sharmila Sengupta, Partha Pratim Mazumdar, Shekhar Chakraborti

**Affiliations:** 1 Division of epidemiology, National Institute of Cholera and Enteric Diseases, P -33, CIT Road Scheme XM, Kolkata, 700010, India; 2 School of Tropical Medicine, 108 C.R. Avenue, Kolkata, 700073, India; 3 Calcutta Medical College, 110 C.R. Avenue, Kolkata, 700073, India; 4 Department of Human Genetics, Indian Statistical Institute, 203 Barrackpore Trunk Road, Kolkata, 700108, India; 5 Division of Virology, National Institute of Cholera and Enteric Diseases, P -33, CIT Road Scheme XM, Kolkata, 700010, India

## Abstract

**Background:**

Prevalence of both cervical cancer and Human Immunodeficiency Virus (HIV) infection are very high in India. Natural history of Human Papilloma Virus (HPV) infection is known to be altered in HIV positive women and there is an increased possibility of persistence of HPV infections in this population. Therefore, this study was conducted to understand the epidemiology and circulating genotypes of oncogenic HPV among HIV positive and negative female population in West Bengal, India.

**Methods:**

In this hospital-based cross-sectional study, 93 known HIV positive females attending a pre-ART registration clinic and 1106 HIV negative females attending a Reproductive and Child Health Care Clinic were subjected to study. Cervical cell samples collected from the study population were tested for the presence of HPV 16, 18 using specific primers. Roche PCR assay was used to detect other specific HPV genotypes in the cervical cells specimens of HIV positive cases only.

**Results:**

Prevalence of HPV 16, 18 among HIV positive females (32.2%; n = 30) was higher than HIV negative females (9.1%; n = 101). About 53% (23/43) of cases with oncogenic HPV were infected with genotypes other than 16, 18 either as single/multiple infections. HPV 18 and HPV 16 were the predominant genotypes among HIV positive and HIV negative subjects respectively. Oncogenic HPV was not found to be associated with age and duration of sexual exposure. But the presence of HIV was found to a statistically significant predictor oncogenic HPV.

**Conclusion:**

The currently available HPV vaccines offer protection only against HPV 16 and 18 and some cross- protection to few associated genotypes. These vaccines are therefore less likely to offer protection against cervical cancer in HIV positive women a high percentage of who were infected with non-16 and non-18 oncogenic HPV genotypes. Additionally, there is a lack of sufficient evidence of immunogenicity in HIV infected individuals. Therefore, prevention of cervical cancer in HIV positive women must be focused towards early detection of oncogenic HPV & cervical cytological abnormality followed by an appropriate treatment.

## Background

Cervical cancer is the most common malignancy among Indian women [1]. India contributes about one fifth of the world cervical cancer burden alone [2]. Various studies have established that oncogenic Human Papilloma Virus (HPV) is associated with almost all cases of cervical cancer [3]. Simultaneously, outside Africa, India has the highest number of people living with Human Immunodeficiency Virus (HIV) [4] that is known to increase the chance of acquiring and persistence of oncogenic HPV in them [5,6]. In immune-competent female subjects, an HPV infection starts appearing in genital tract following first sexual exposure and is understood to be cleared off with increase of age and mostly by the age of 30 year as revealed by some studies. [7,8] But, the natural history of HPV infection is known to be altered in persons infected with the HIV and there is an increased possibility of persistence of HPV infections in this population [5,6]. Persistent oncogenic HPV infection increases their risk of having cervical precancerous and cancerous lesions [5,6]. Thus HIV positive females have an increased chance of developing cervical carcinoma which is considered to be an AIDS indicating condition. Little data is available to understand the existence and magnitude of problem of oncogenic HPV in HIV infected female population in Southeast Asian countries, where India alone contributes the largest number of HIV infected individuals. This data is necessary to understand the epidemiology of oncogenic HPV among female population with and without HIV and its additional infectivity caused by HIV infection if any. This is also required to understand the prevalent strains of various oncogenic HPV that are prevalent in India both among HIV infected and uninfected individuals. This might justify the use of a currently available HPV vaccination as a step towards primary prevention of cervical cancer in India. Hence, this study was conducted in female population (with and without HIV infection) of West Bengal, eastern India to understand the epidemiology of oncogenic HPV.

## Methods

It was a hospital-based cross-sectional study. The study aimed to compare the prevalence of HPV-16 and 18 among HIV positive and HIV negative subjects with associated socio-demographic variables. The former was selected from the patients attending the pre-ART registration clinic of School of Tropical Medicine Hospital (only state hospital offering ART to eligible HIV infected people in southern part of the state), Kolkata. A total of 93 ART naïve, known HIV positive females participated voluntarily in this study. Written informed consent was obtained from all of them. Interview with a pre-tested questionnaire was used to collect their information on socio-demography and relevant sexual behavior. Interview was followed by collection of cervical cells from external os and adjoining ecto-cervical area with the help of a cervical cyto-brush and disposable vaginal speculum to avoid transmission of STIs among them.

A total of 1106 HIV negative females attending a Reproductive and Child Health (RCH) Care Clinic in a Southern part of West Bengal area were taken as controls. The controls had similar socio-economic status as that of cases as evidenced by their income, occupation & education. Informed consent was taken from all of them. Brief interview was followed by collection of cervical cells (as described in collection of specimen from cases). The major reason for their attending the clinic was for routine reproductive health care counseling or for getting treatment for some common gynecological ailments. Pregnancy was excluded from both cases and control subjects. Collected cervical specimens were dipped into cold phosphate-buffered saline (PBS) and transported to the laboratories of the Department of Human Genetics, Indian Statistical Institute (ISI) and National Institute of Cholera & Enteric Diseases (NICED), Kolkata, for HPV DNA testing. At ISI, HPV 16, 18 specific primers were used to detect these 2 genotypes (most common among Indian females [9]) in cervical samples from both cases and controls. At NICED laboratory, Linear Array HPV Genotyping Test (Roche) was used to detect other specific HPV genotypes in the cervical cells specimens of cases only. Because of cost constraints, Roche PCR test was done only among samples collected from HIV positive cases. HPV target DNA for PCR amplification was prepared using Amplitude Liquid Media Extraction Kit. Amplification of target DNA by PCR using HPV primers was followed by hybridization of the amplified products. The assay amplifies HPV genotypes 6, 11, 16, 18, 26, 31, 33, 35, 39, 40, 42, 45, 51, 52, 53, 54, 55, 56, 57, 58, 59, 66, 68, 73, 82, 83, and 84. The HPV genotypes considered high risk included 16, 18, 26, 31, 33, 35, 39, 45, 51, 52, 55, 56, 58, 59, 68, 73 and 82. [10,11].

Statistical analyses were performed using SAS (Version 9.1 for Windows; SAS Institute Inc., NC, USA) software. Chi-Square Test for Homogeneity and two sample T test were performed to compare demographic variables of the independently chosen study samples. The associations of potential risk factors with HIV/HPV infection were estimated by means of odds ratios (OR) and 95% confidence intervals (95% CI). All reported *p*-values are two-sided. The significance level for the predictors to stay in the model was 0.05.

Ethical clearance was obtained from the ethical committee of the National Institute of Cholera & Enteric Diseases (NICED) before initiation of the study.

## Results

A total of 93 HIV positive women and 1106 HIV negative women participated in this study. Cases and controls did not differ significantly with respect to mean age, religion and parity (Table 1). However, differences in education, mean age of marriage and duration of sexual life between cases and controls were statistically significant (P-value < .001).

**Table 1:** Socio-demographic variables of study population according to HIV sero-status

Variable	HIV positive women (n = 93)	HIV negative women (n = 1106)	P value	Comments
Mean age				

	29 yrs	30 yrs	P-value > .001	There is not convincing evidence that HIV positive and negative samples differ with respect to mean age

**Religion**				

Hindu	82%	65%	P-value > .001	There is not convincing evidence that HIV positive and negative samples differ with respect to religion
Muslim	18%	31%		
Christian	0%	4%		

**Education**				

Illiterate	52%	25%	P-value < .001	HIV positive and negative samples differ with respect to literacy status
Primary	25%	64%		
Secondary	13%	6%		
Higher Secondary & College	11%	5%		

**Mean age of marriage**				

	18 years	16 years	P-value < .001	HIV positive and negative samples differ with respect to mean age of marriage

**Mean duration of sexual life**				

	10 years	13 years	P-value < .001	HIV positive and negative samples differ with respect to mean duration of sexual life

**Life time sexual partners**				

1	96%	99%	P-value could not be calculated because one of the expected counts was less than 5	
2	4%	1%		

**Parity**				

0	6%	6%	P-value > .001	There is not convincing evidence that HIV positive and negative samples differ with respect to parity
1-2	51%	56%		
> 2	43%	38%		

Among HIV positive study population, prevalence of HPV was found to be 56% (n = 52), prevalence of oncogenic HPV was 46.2% (n = 43) and prevalence of HPV 16 and/or 18 was 32.2% (n = 30). Among HIV negative study population prevalence of HPV 16 and/or 18 was 9.1% (n = 101) [OR = 4.7; CI: 2.8 - 7.9]. Thus, HIV positive females were almost 5 times more at risk of acquiring oncogenic HPV co-infection than HIV negative females.

**Figure 1:**
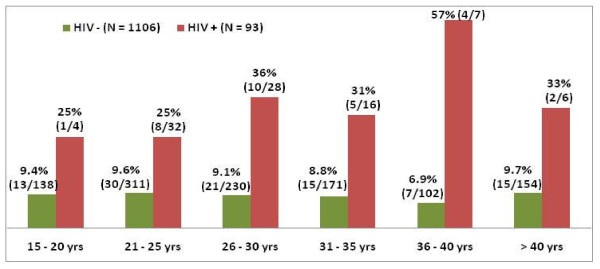
**Distribution of HPV-16 & 18 among women with and without HIV infection by age**.

Figure 1 shows age-wise distribution of oncogenic HPV (indicated by HPV genotype-16 and/or 18) among cases and controls. It shows that, in controls, prevalence of HPV 16 and 18 is almost constant (approx. 9%) across different age groups. But, in cases, prevalence of HPV shows a rising trend till 40 years after which prevalence decreases. Prevalence of HPV is higher among cases in each age group compared to controls.

**Figure 2:**
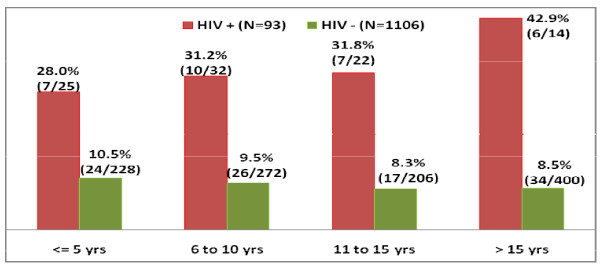
**Distribution of HPV-16 & 18 in relation to sexual duration in HIV+ve & HIV-ve subjects**.

Figure 2 describes distribution of oncogenic HPV in relation to duration of active sexual life. It is evident that among controls prevalence of oncogenic HPV shows a slightly decreasing trend with increase in duration of sexual life whereas among cases it shows a rising trend. Secondly, prevalence of oncogenic HPV is much higher in cases compared to controls for any particular duration of active sexual life.

**Figure 3:**
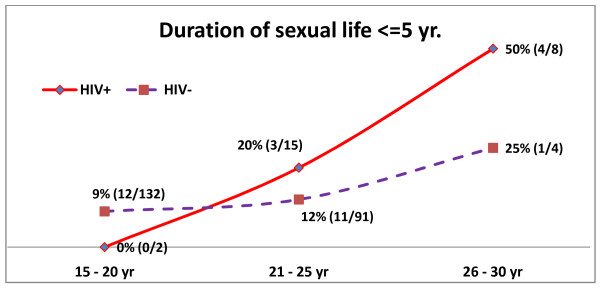
**Age-wise distribution of HPV-16 & 18, when duration of sexual act < = 5 yr**.

**Figure 4:**
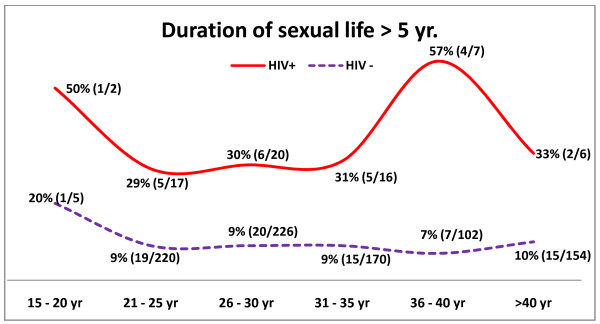
**Age-wise distribution of oncogenic HPV-16 & 18 when duration of sexual act > 5 yr**.

Figure 3 shows age-wise distribution of HPV among cases and controls with duration of sexual life of 5 year or less. Interestingly, in this group HPV prevalence showed a steadily increasing trend with age among both HIV positive and negative females. Whereas, when duration of sexual life is more than 5 years (figure 4), prevalence of HPV shows a decreasing trend with age till 30 years for both cases and controls. Beyond 30 years, the prevalence shows an almost parallel trend among controls but, it steadily increases among cases till 40 years beyond which it again declines.

On univariate analysis, the significant predictors of HPV infection were HIV, age, and duration of sexual life. Though other demographic predictors such as education level, religion and socio-economic status have been found to be confounding factors in other published study, these factors did not seem to be significant confounders in our study. Next, both bivariable and multivariable logistic regression analysis were conducted to further analyze data. In bi-variable logistic regression Wald chi-square test statistic and p value was used. In bi-variable models HIV was a significant predictor of HPV (OR = 4.8, CI 2.9 - 7.8, p < 0.05). Neither age (OR = 0.9, CI 0.9 - 1.0, p > 0.5) nor duration of sexual life (OR = 0.9, CI 0.9 - 1.0, p > 0.05) were found to be statistically significant as predictors of HPV. Next, we conducted multivariable logistic regression to find out if multicollinearity or synergism existed between the three predictors (HIV, age, and duration of sexual life). Chi square (likelihood ratio) and p value were used as test statistics. After adjusting for age and duration of sexual life, the OR for HIV was 4.9 (CI 2.9 - 8.0, p < 0.05) i.e. the odds of having HPV infection were 4.9 times higher among HIV sero-positive women than HIV sero-negative women. Age and duration of sexual life were not statistically significant predictors of HPV after adjusting for HIV status and duration of sexual life, and HIV status and age, respectively (Age: OR = 0.9, CI 0.9 - 1.0, p > 0.05; Duration of sexual life: OR = 1.01, CI 0.9 to 1.1, p > 0.05).

**Figure 5:**
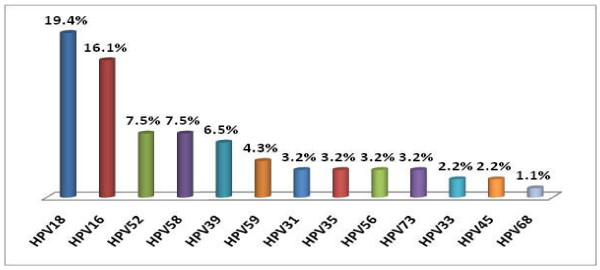
**Distribution of oncogenic HPV genotypes among HIV infected subjects tested by Roche kits (n = 93)**.

Distribution of the oncogenic HPV in HIV infected subjects is shown in Figure 5. It is evident that oncogenic genotype-18 was the predominant pathogen (19%) followed by that of type-16 (16%) in HIV infected study subjects. Strikingly, prevalence of HPV 18 among the controls was found to be very low (0.9%; n = 10). Among other genotypes, HPV 52, 58 and 39 were observed in a sizable portion of cases. This is a matter of concern.

About 17% (n = 15) of the cases were infected by multiple oncogenic strains of HPV of which 8 were infected with dual genotypes, 6 were infected with triple genotypes and only 1 was infected with 4 genotypes. HPV 16 and/or 18 were present in 13 out of 15 multiple genotype infected cases.

## Discussion

In this study it was observed that the prevalence of HPV 16 and or18 among HIV negative female population to be 9.1% which is comparable to findings from various other studies conducted in India. [9] The study demonstrates significantly higher prevalence of oncogenic HPV (all genotypes) and HPV 16/18 among HIV positive cases compared to HIV negative controls. Two earlier studies from western and southern India and studies conducted in other parts of the world have also documented similar findings. [12-16]. This study has also documented that HIV positive females are at increased risk of getting infected with oncogenic HPV than the HIV negative females irrespective of age and sexual life duration and risk becomes higher when age more than 30 year and sexual exposure duration more than 5 year. Probably this is related with declining body immunity as seen in more advanced HIV infected cases.

Generally HPV prevalence in women shows a peak at younger ages and then declines over the age of 30 years [7,8]. Even in studies among sex workers, a population which is highly exposed to HPV, Kjaer et al. and Sarkar et al observed a considerably decreasing trend in HPV prevalence with age in spite of continuous high levels of sexual activity. [17,18] This is probably due to development of acquired immunity to HPV infection following repeated exposure. But, in this study, it was found that the prevalence of HPV 16/18 to be almost constant across different age groups among the controls. Similar findings were noted in other studies on HPV epidemiology in the Indian Subcontinent where oncogenic HPV prevalence did not vary significantly across age groups in India. [19-23] There was no clear peak of HPV prevalence in young women. However it should be noted that in these studies and also in the current study, subjects included were primarily married women. This was because in India genital tract sampling of an unmarried woman is associated with cultural and social taboo. Socio-cultural practices specifically related to Indian married women might have played a role in the lack of a peak in HPV prevalence among younger age groups. In the present study most of the cases reported to have monogamous relationship (96%, n = 89). Number of life time partners, sexual behavioral patterns, number of pregnancies, genital hygiene, presence of other STDs, socio-economic conditions, literacy status, underrepresentation of teenagers in the study samples etc. might have influenced the low clearance of incident HPV infections and may be responsible for the constant, steady prevalence of HPV in different age groups in India. On the other hand, frequent re- infection/reactivation due to multiple sex-partners, increased frequency of sex etc. might have helped in the development of immunity against HPV among sex workers in the study by Sarkar et al. [18] which included young unmarried women. An in-depth cohort study from this region is required to understand this.

In HIV positive females, prevalence of HPV 16 and/or18 showed a rising trend till 40 years after which it declined. Since this was a cross-sectional study it was impossible to determine whether HPV infection followed HIV or otherwise. But earlier studies have shown that presence of HIV infection facilitates the development and persistence of HPV infection. This may be because HIV infection lowers body immunity. So, co-infection with HIV can be a probable explanation for acquisition of new HPV infections later in life, delayed clearance of HPV with age and reactivation of latent infections acquired earlier in life due to a gradual loss of immunity. The decline of HPV prevalence beyond 40 years may be influenced by less number of subjects caused by death due to AIDS, increased immunity following receiving ART etc.

Duration of sexual activity had opposite influence on HPV 16 and/or18 prevalence among HIV positive and negative subjects. HPV showed rising trend with increase in duration of sexual activity in HIV positive females whereas it showed a decline with increase in duration of sexual activity among HIV negative females. But, when adjusted for both age and duration, HPV prevalence showed a steady rise till 30 years of age in cases and controls when duration of active sexual life was less than equal to 5 years. However, when duration was more than 5 years, HPV declined with age till 30 years in both HIV positive and negative females. This may probably be due to development of immunity against HPV with higher duration of sexual life. So, duration of sexual life is perhaps a greater determinant of development of immunity and clearance of HPV than age alone till 30 years even in the presence of HIV. After 30 years, even with higher duration of sexual life HPV showed a constant prevalence with increase in age in HIV negative subjects whereas in the HIV positive group HPV increased with age till 40 years beyond which it again declined. In this study we were unable to compare these trends with those in population above 30 years of age but duration of sexual life less than equal to 5 years since none of the subjects belonged to this group. However, in multivariate analysis neither age nor duration of sexual life were found to be significant predictors of HPV infection. HIV co-infection was the only significant predictor of HPV infection and HIV positive women were almost five times more likely to be infected by oncogenic HPV compared to HIV negative women.

Previous studies in India have reported circulating strains of oncogenic genotypes other than HPV 16 and/or18 in the general population [9]. But adequate data is not available regarding oncogenic strains of HPV other than HPV16 and/or18 in HIV infected Indian women especially from Eastern India. Due to resource constraints we were only able to perform a PCR assay of cervical specimens obtained from HIV positive subjects. Our findings document the presence of almost all oncogenic genotypes of HPV in HIV positive females. Among controls HPV 18 showed a much lower prevalence than HPV 16 (0.9% vs. 9%). Similar findings were noted in other studies from different parts of India. [9] However, among HIV +ve subjects, HPV 18 showed the highest prevalence followed by HPV 16. This predominance of HPV 18 among HIV positive women in a setting where HPV 16 is the most prevalent genotype among HIV negative women requires further exploration. Demographic differences in the study population in terms of education, age of marriage and duration of sexual life does not seem to influence transmission of different genotypes. Unfortunately cytological tests were not a part of the study due to lack of resources. Therefore, further research needs to be done to see if the predominance of HPV 18 in HIV positive subjects was due to an increased number of cases of adeno-squamous lesions in this population. Another possible cause may be higher transmission potential of HPV-18 among HIV+ve subjects. Strikingly, about 53% (23/43) of cases with oncogenic HPV were infected with genotypes other than 16 and/or18 either as single/multiple infections. In a review article by McKenzie et al. HIV infected women in different geographic regions appeared to be infected with less prevalent types of HR-HPV as compared to the general population. [24] High-risk HPV types were isolated from 97% of cervical cancer cases, and HPV-16/18 were found in 80% of cervical neoplasia in India [9,19-23]. This shows that oncogenic HPV other than HPV 16/18 plays a substantial role in the development of cervical cancer especially in HIV infected women.

## Conclusion

With the advent of ART, HIV positive women tend to live longer. Since cervical cancer generally develops in older age groups, therefore in the era of ART, there is an increased chance of cervical cancer cases among HIV positive women because of longer survival period. Information on interaction between HIV and HPV is therefore important to understand individual influence of each virus against the other. The currently available HPV vaccines offer protection only against HPV 16 and 18 and some cross-protection to few associated genotypes. Moreover, there is lack of evidence of immunogenicity in HIV infected individuals following receiving HPV 16/18 vaccines. These vaccines are therefore less likely to offer protection against cervical cancer in HIV positive women who harbor a high percentage of other oncogenic HPV genotypes. Therefore in HIV positive women early detection of oncogenic HPV & cervical cytological abnormality followed by an appropriate treatment is possibly the best approach at present.

## Competing interests

The authors declare that they have no competing interests.

## Authors' contributions

KS: made substantial contributions to conception, design, implementation and overall supervision of all the steps of this study as Principal investigator. RP: made substantial contributions to acquisition, analysis and interpretation of data and have been involved in drafting the manuscript. BB: have made substantial contributions to acquisition and editing of data. BS: have made substantial contributions to acquisition and editing of data. SB: have made substantial contributions to acquisition and editing of data. SS: carried out the HPV DNA testing using HPV 16, 18 specific primers. PPM: coordinated and supervised laboratory work including quality assurance. SC: carried out the HPV DNA testing using HPV 16, 18 specific primers and also conducted Linear Array HPV Genotyping Test (Roche) as chief of laboratory.

## Pre-publication history

The pre-publication history for this paper can be accessed here:

http://www.biomedcentral.com/1471-2334/11/72/prepub
